# Effects of Temperature and Strain Rate on the Fracture Behaviors of an Al-Zn-Mg-Cu Alloy

**DOI:** 10.3390/ma11071233

**Published:** 2018-07-18

**Authors:** Yue Guo, Mingxing Zhou, Xingdong Sun, Long Qian, Lijia Li, Yingjie Xie, Zeyang Liu, Di Wu, Liguo Yang, Tong Wu, Dan Zhao, Jinguo Wang, Hongwei Zhao

**Affiliations:** School of Mechanical Science and Engineering, Jilin University, Changchun 130025, China; yueguo15@mails.jlu.edu.cn (Y.G.); zhoumx15@mails.jlu.edu.cn (M.Z.); xdsun15@mails.jlu.edu.cn (X.S.); qianlong15@mails.jlu.edu.cn (L.Q.); llj15@mails.jlu.edu.cn (L.L.); xieyj16@mails.jlu.edu.cn (Y.X.); zeyang16@mails.jlu.edu.cn (Z.L.); wud15@mails.jlu.edu.cn (D.W.); yanglg15@mails.jlu.edu.cn (L.Y.); wutong16@mails.jlu.edu.cn (T.W.); zhaodan16@mails.jlu.edu.cn (D.Z.); jgwang@jlu.edu.cn (J.W.)

**Keywords:** quasi-static tensile deformation, Al-Zn-Mg-Cu alloy, plastic deformation, fracture morphology, microstructure

## Abstract

Effects of temperature and strain rate on the fracture behaviors of an Al-Zn-Mg-Cu alloy are investigated by isothermal uniaxial tensile experiments at a wide range of temperatures and strain rates, from room temperature (RT) to 400 °C and from 10^−4^ s^−1^ to 10^−1^ s^–1^, respectively. Generally, the elevation of temperature leads to the increasing of elongation to fracture and the reduction of peak stress, while higher strain rate results in the decreasing of elongation to fracture and the increasing of peak stress. Interestingly, we found that the coefficient of strain rate sensitivity (*m*-value) considerably rises at 200 °C and work of fracture (*W_f_*) fluctuates drastically with the increase of strain rate at RT and 100 °C, both of which signify a non-uniform and unstable deformation state below 200 °C. A competition of work hardening (WH) and dynamic recrystallization (DRX) exists at 200 °C, making it serve as a transitional temperature. Below 200 °C, WH is the main deformation mechanism of flow stress, and DRX dominates the flow stress above 200 °C. It has been found that from RT to 200 °C, the main feature of microstructure is the generation of dimples and microvoids. Above 200 °C, the coalescence of dimples and microvoids mainly leads to the failure of specimen, while the phenomenon of typically equiaxed dimples and nucleation appear at 400 °C. The observations of microstructure are perfectly consistent with the related macroscopic results. The present work is able to provide a comprehensive understanding of flow stress of an Al-Zn-Mg-Cu alloy at a wide range of temperatures and strain rates, which will offer valuable information to the optimization of the hot forming process and structural design of the studied alloy.

## 1. Introduction

As one of the most crucial engineering materials, the high-strength Al-Zn-Mg-Cu alloy has been widely used in marine, aeronautics, and automobile industries due to its excellent strength-to-weight ratio and resistance to stress corrosion cracking behavior. The introduction of elements, such as Zn, Mg, and Cu, can effectively increase its strength at room temperature (RT). However, high contents of alloying elements may simultaneously weaken the hot workability of the alloy [1]. It has been widely acknowledged that deformation mechanisms of work hardening (WH) [2,3], dynamic recrystallization (DRX) [4,5], and dynamic recovery (DRV) [6,7] can result in different deformation behavior of Al-Zn-Mg-Cu alloy. Therefore, the problem of how to enhance the mechanical properties and optimize the formability of Al-Zn-Mg-Cu alloy has become a research hotspot in recent years, and some researches have been made to explore its hot deformation behaviors at quasi-static strain rate [8,9,10]. Zhou, M et al. [1] performed uniaxial tensile tests under temperature of 340–460 °C and strain rate of 10^−3^–10^−2^ s^−1^. It has been found that the obtained true stress-true strain curves include four different stages, namely elastic stage, uniform deformation stage, diffusion necking stage, and localized necking stage. Shojaei, K. et al. [11] compared influences of temperature and strain rate on annealed and cold rolled 7075 aluminium, at strain rates and temperatures ranging from 10^−3^ s^−1^ to 10^−1^ s^−1^ and 200 to 350 °C, respectively. They found that flow behavior of both annealed and cold rolled 7075 aluminium can be strongly affected by temperature and strain rate, and the influence on annealed specimen is likely to be less remarkable. Mishra, R.S. et al. [12] studied the effects of temperature on superplasticity of 7075 aluminium alloy processed by friction stir welding (FSW), and revealed that the optimum superplasticity could be achieved at 490 °C. Meanwhile, the complex variation of microstructure occurring during hot processing is able to affect mechanical properties of the alloy. Heretofore, investigating microstructural evolution would be an effective method to explore the underlying mechanism of the macroscopic fracture [13,14,15]. Wang, L. et al. [5] found that recrystallized grains and a high proportion of high-angle grain boundaries at high temperature can lead to grain boundary sliding (GBS), which results in a large elongation of 200% of twin roll casted (TRC) 7075 aluminum alloy at strain rate of 5 × 10^−3^ s^−1^and temperature of 450 °C. El-Magd, E. et al. [16] asserted that under tensile loading, nucleation, growth, and the coalescence of microvoids dominates the failure of AA7075 alloy. According to Taheri-Mandarjani, M. et al. [17], the decline of ductility at 350 °C and at strain rates of 10^−2^ and 10^−3^ s^−1^ is mainly due to the brittle Fe-rich phases during the deformation process.

Although some researches have focused on the flow behaviors of Al-Zn-Mg-Cu alloy, the selected range of temperatures and strain rates can be relatively narrow. The investigation of the mechanical deformation mechanism of the studied alloy below 300 °C and 10^−3^ s^−1^ has always been neglected, thus failing to provide an overall insight into the understanding of deformation behaviors influenced by temperature and strain rate. More comprehensive experimental research of the deformation behavior of Al-Zn-Mg-Cu alloy at wide temperature and strain rate ranges is still required, so that the mechanical deformation mechanism of the alloy can be thoroughly understood. Thus, a wide range of temperatures (RT—400 °C) and strain rates (10^−4^–10^−1^ s^−1^) have been chosen in this study. Their effects on the fracture behavior and microstructure evolution mechanism of Al-Zn-Mg-Cu alloy are discussed in detail by carrying out isothermal uniaxial tensile tests. The fracture surface of specimen has been observed by scanning electron microscope (SEM), and the main feature of microstructure at different temperature ranges has been discussed with respect to its deformation mechanism. Furthermore, strain rate sensitivity activated by thermal condition has been deeply analyzed, and we found that 200 °C can serve as a transitional temperature for the hot deformation mechanism of the alloy. From 200 °C, the deformation transfers into a uniform and stable state. Finally, the coupling effect of temperature and strain rate has been analyzed. In this way, the hot deformation mechanism of Al-Zn-Mg-Cu alloy at quasi-static strain rate can be thoroughly understood, which will offer more valuable and reliable information to the optimization of the hot forming process, structural design, and mechanical properties of the alloy.

## 2. Materials and Methods

**Figure 1:**
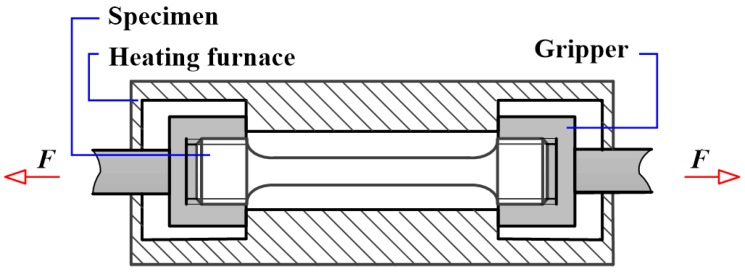
Schematic diagram of the isothermal uniaxial tensile experiment.

The adopted specimen in this study is a typical hot-rolled Al-Zn-Mg-Cu alloy (6.07% Zn, 3.39% Mg, 1.57% Cu, 0.57% Si, and 0.31% Cr). Cylindrical specimens, with a gauge length of 36 mm and a diameter of 5 mm, were machined along the rolling direction. Uniaxial tensile tests were carried out at five different deformation temperatures (RT, 100 °C, 200 °C, 300 °C, and 400 °C) and four different strain rates (10^−4^ s^−1^, 10^−3^ s^−1^, 10^−2^ s^−1^, and 10^−1^ s^−1^), which were conducted by Zwick Z250 universal testing machine (Zwick, Ulm, Germany). The schematic diagram of the isothermal uniaxial tensile experiment is shown in Figure 1. Apart from experiments carried out at RT, specimens were heated up to corresponding deformation temperature and held isothermally for 20 min prior to loading in order to eliminate the thermal gradient. Then, experiments were performed at designed temperatures and strain rates. Repetitive experiments have been made to ensure the reliability of experiment results.

After isothermal uniaxial tensile experiments, fracture surfaces of specimens were examined utilizing SEM (ZEISS EVO18 Special Edition, Oberkochen, Germany) to investigate effects of various deformation parameters on the microstructure of the studied Al-Zn-Mg-Cu alloy.

## 3. Results and Discussion

### 3.1. Flow Stress

The engineering stress and engineering strain are calculated by Equations (1) and (2):

(1) $$\sigma =F/A_{0}$$

(2) $$\varepsilon =\Delta l/l_{0}$$

where *σ*, *ε*, *F*, *A*_0_, ∆*l*, and *l*_0_ represent engineering stress, engineering strain, tension force, the initial cross section area of specimen, elongation of specimen in the direction of tensile, and the initial gauge length, respectively.

**Figure 2:**
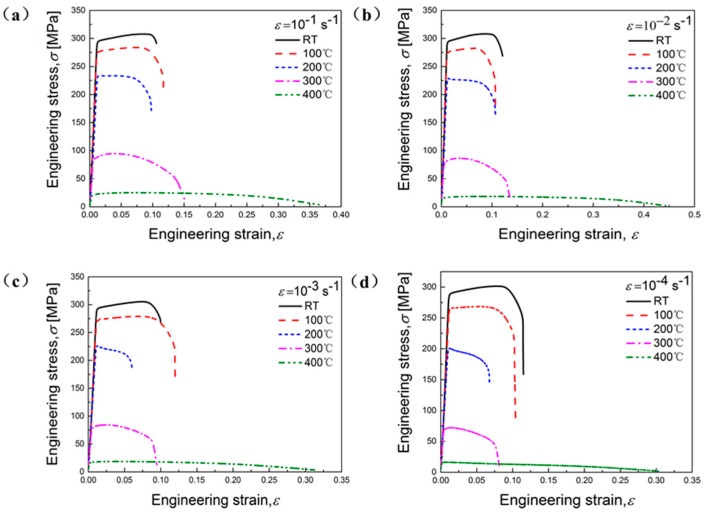
Engineering stress versus strain curves of the studied Al-Zn-Mg-Cu alloy for tension at various strain rates: (**a**) 10^−1^ s^−1^; (**b**) 10^−2^ s^−1^; (**c**) 10^−3^ s^−1^; (**d**) 10^−4^ s^−1^.

**Figure 3:**
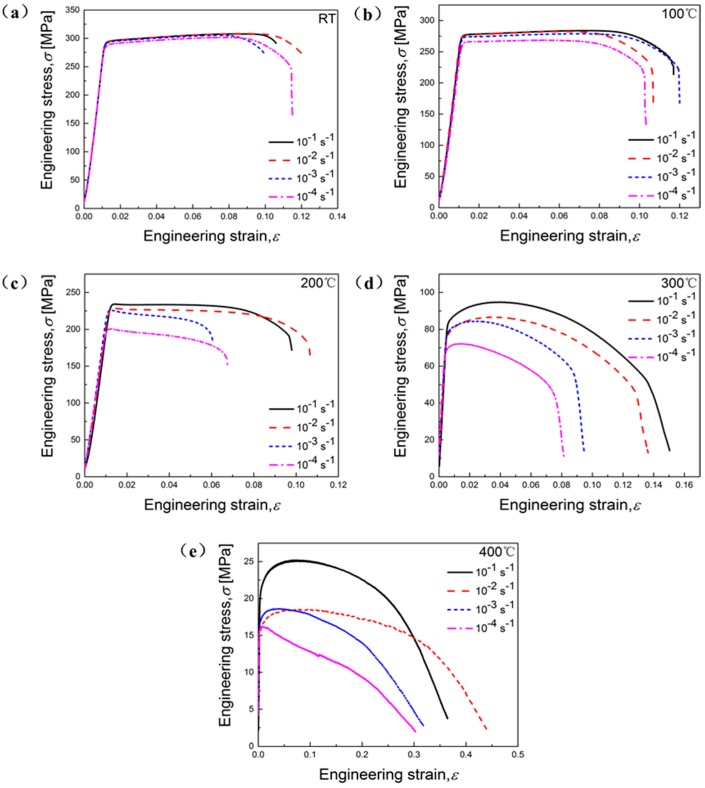
Engineering stress versus strain curves of the studied Al-Zn-Mg-Cu alloy for tension at various temperatures: (**a**) RT; (**b**) 100 °C; (**c**) 200 °C; (**d**) 300 °C; (**e**) 400 °C.

It is obviously that both deformation temperature and strain rate have significant influences on flow stresses of the studied alloy (Figure 2 and Figure 3). As a general trend, fracture strain rises with the elevation of temperature at each strain rate in Figure 2. It can be found that flow stress rises with the increasing of strain rate at given temperature, as depicted in Figure 3, mainly due to the enhanced deformation storage energy [5].

Moreover, at RT and 100 °C, curves at each strain rate show that the stress rises with the increase of plastic deformation until reaching the peak stress, indicating an evident WH (Figure 2 and Figure 3a,b). This means that at RT and 100 °C and from 10^−4^ s^−1^ to 10^−1^ s^−1^, the WH effect is stronger than the softening mechanism resulting from DRV. At 200 °C and each strain rate, it can be observed that there exists an abrupt reduction of stress after reaching peak stress, which shows a competitive process of DRX, WH, and DRV (Figure 2 and Figure 3c). Above 200 °C, the stress declines steadily after the reach of peak stress, demonstrating an obvious thermal softening phenomenon, because the DRV and DRX activated by temperature dominates the deformation process (Figure 2 and Figure 3d,e). In Figure 3, it can be seen that the flow stress of the studied alloy become more sensitive to the variation of strain rate from 200 °C. Therefore, 200 °C can be defined as a transition stage, below which the WH dominates the deformation, and above which the DRX and DRV mechanisms induced by high temperature mainly influence the deformation behaviors.

### 3.2. Effects on Mechanical Properties of Al-Zn-Mg-Cu Alloy

In general, the plastic deformation capability of materials can be measured by the elongation to fracture, which can be calculated by Equation (3):

(3) $$\delta =\frac{l_{k}-l_{0}}{l_{0}}\times 100\%.$$

where *δ* and *l_k_* represent elongation to fracture and the fractured gauge length, respectively.

**Figure 4:**
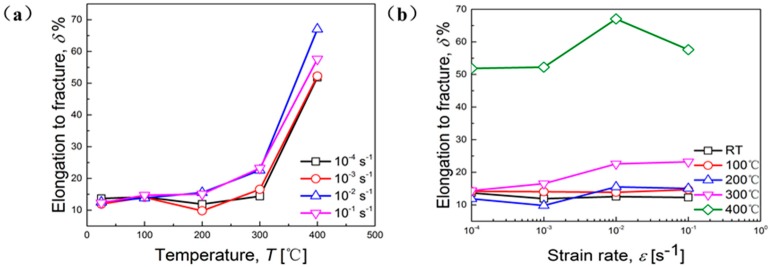
Elongation to fracture as a function of: (**a**) Temperature; (**b**) strain rate.

Figure 4 depicts the elongation to fracture under various deformation conditions of the studied material. Apparently, values of elongation to fracture increase with the elevation of temperature, especially above 300 °C (Figure 4a). This observation is mainly due to more refined microstructure and the occurrence of DRX caused by high temperature, which will be further verified by subsequent SEM analyses. The ductility of alloy at higher temperature is closely related to DRX, which can be affected by influencing the variation of microstructures [18]. DRX is difficult to induce at relatively low temperature, which eventually results in small elongation to fracture. Sunter, B.J. et al. [19] proposed that the ductility behavior of copper alloy can be enhanced because of DRX. In Figure 4b, values of elongation to fracture increase with the enhancement of strain rate, especially at temperatures above 200 °C. From Figure 4a, it can be also seen that the variation of elongation to fracture to strain rates becomes more sensitive above 200 °C, mainly due to a fine-grained microstructure led by high temperature.

**Figure 5:**
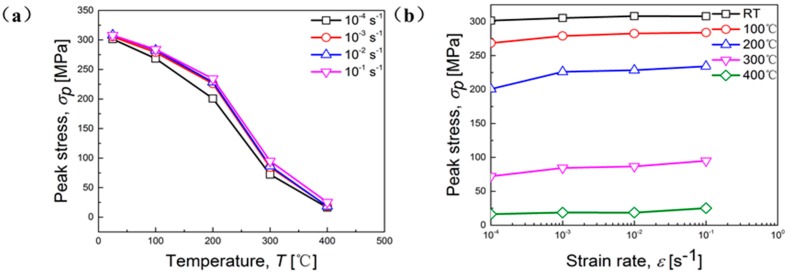
Peak stress as a function of: (**a**) Temperature; (**b**) strain rate.

The changes of peak stress at different temperatures and strain rates are presented in Figure 5. It is apparent that the peak stress declines with the increasing of temperature and the decreasing of strain rate. This phenomenon can be attributed to the coupling effect of temperature and strain rate. Higher temperature facilitates the mobility of nucleation and dislocation annihilation, eventually resulting in the occurrence of DRX, whose typical features are nucleation, grain growth, and the formation of equiaxed microstructure [20]. All of these features can be observed in the following fracture images shot by SEM. On the other hand, the thermal activation process and dynamic softening mechanism activated by high temperature can accelerate dislocation motion and lead to the reduction of peak stress [17,21,22,23]. Lower strain rate is able to provide a longer time for the recovery and accumulation of deformation energy.

### 3.3. Strain Rate Sensitivity

**Figure 6:**
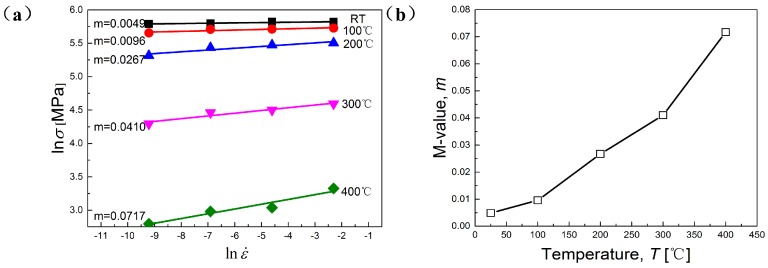
The variations of: (**a**) natural logarithm of flow stress vs. strain rate; (**b**) strain rate sensitivity vs. temperature.

The strain rate sensitivity coefficient (*m*-value) demonstrates the transfer and diffusion capacity of necking. At high deformation temperature, the plastic deformation ability of materials enhances with the increasing of *m*-value [16,22]. *m*-Value can be calculated by Equation (4), which means that *m*-value can be calculated by the slope of curves at constant temperatures and strain rates by linear fitting method [1,9,17,24,25,26,27]. Figure 6 demonstrates the linear fitting results of *m*-value at different temperatures and the tendency of its variation with the elevation of temperature.

(4) $$m={\frac{\partial \ln\sigma }{\partial \ln\dot{\varepsilon }}|}_{\varepsilon ,T}$$

As depicted in Figure 6b, *m*-value shows strong dependence on temperature, mainly due to the thermal activated mechanism induced by high temperature [28]. In other words, the elevation of temperature remarkably accelerates the diffusion softening process. It is widely acknowledged that high temperature is able to improve the necking diffusion ability, and that better transfer and diffusion ability of necking can bring a more uniform and stable plastic deformation process [29].

Interestingly, it has been found that *m*-value rises drastically from 200 °C, which means that mechanical properties of the studied alloy become more sensitive to the variation of strain rate. This result perfectly accords with the analyses mentioned above. Taheri-Mandarjani, M. et al. [17] found that the post-uniform (beyond necking) elongation of the material to fracture is controlled by *m*-value. Therefore, a higher *m*-value may benefit the delay of the occurrence of localized deformation and eventually improve the ductility of the studied alloy. The correspondingly low *m*-value at RT and 100 °C indicates a non-uniform and instable deformation process, mainly caused by interactions between dislocations and a slow rate of solute diffusion [16]. It can be deduced that from 200 °C, DRX and the dynamic softening mechanism have been activated and accelerated, making the alloy become more sensitive to the change of strain rate at quasi-static level.

### 3.4. Work of Fracture

The work of fracture (*W_f_*) can be calculated by integrating the area below the nominal stress-strain curve:

(5) $$W_{f}=\int_{0}^{\varepsilon_{L}}\sigma d\varepsilon$$

where *ε_L_* represents the elongation to fracture and σ represents the nominal stress.

**Figure 7:**
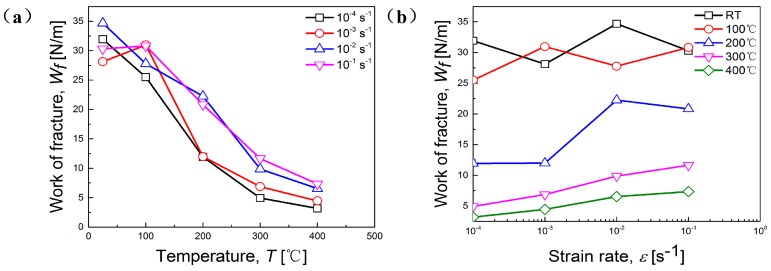
Work of fracture (*W_f_*) versus (**a**) temperature at different strain rates; (**b**) strain rate at different temperatures.

As presented in Figure 7, *W_f_* reduces with the elevation of temperature (Figure 7a). It can be seen that at RT and 100 °C, *W_f_* fluctuates remarkably with the variation of strain rate, but the difference of *W_f_* between 10^−4^ s^−1^ and 10^−1^ s^−1^ is very small, indicating a non-uniform and instable deformation. From 200 °C, *W_f_* rises steadily with the increasing of strain rate at each given temperature, and besides, the largest difference of *W_f_* can be observed at 200 °C (Figure 7b). The above phenomenon perfectly accords with the explanation of *m*-value. It is said that *W_f_* is a comprehensive measure containing both flow stress and damage characteristics, and *W_f_* is influenced by the temperature- and rate-sensitivity of the flow stress and the inherent damage mechanism [22]. Thus, it can be deduced that 200 °C is a transitional temperature for plastic deformation of the studied alloy from a fluctuant state to a more uniform and stable state.

### 3.5. Analyses of Fracture Morphology

**Figure 8:**
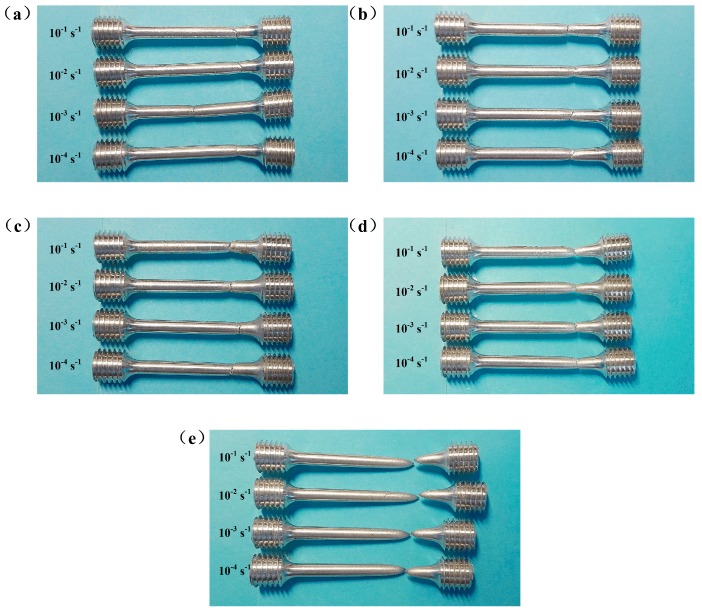
Fracture specimens at temperatures of: (**a**) RT; (**b**) 100 °C; (**c**) 200 °C; (**d**) 300 °C; (**e**) 400 °C.

Fracture specimens at various temperatures and strain rates are shown in Figure 8. Obviously, all specimens exhibit apparent necking and are typical cup-and-cone fracture. This phenomena is more remarkable with increasing of temperature (Figure 8e). As mentioned before, the *m*-value of the studied material increases with the elevation of temperature (Figure 6b), indicating that the transfer and diffusion ability of necking enhances with the elevation of temperature. Therefore, the obvious necking behavior at high temperature, shown in Figure 8, is consistent with results of *m*-value (Figure 6b) and elongation to fracture (Figure 4a).

**Figure 9:**
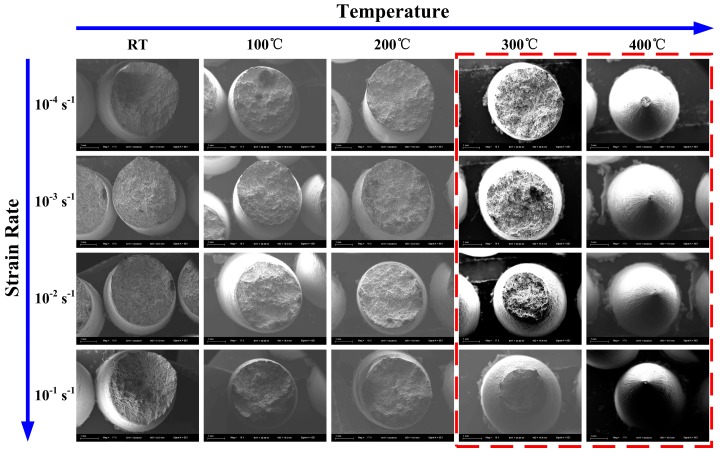
Fracture surface of specimens at various temperatures (RT—400 °C) and strain rates (10^−1^–10^−4^ s^−1^).

In order to investigate deformation mechanisms and the fracture behavior more deeply, selected specimens were further observed by SEM. The fracture surface of specimens at various temperatures and strain rates are presented in Figure 9. Apparently, with the increase of temperature, necking behavior is more noticeable. Furthermore, at 300 °C and 400 °C, with the elevation of strain rate, necking behavior can be more remarkable.

#### 3.5.1. Effects of Temperature on Fracture Morphology

**Figure 10:**
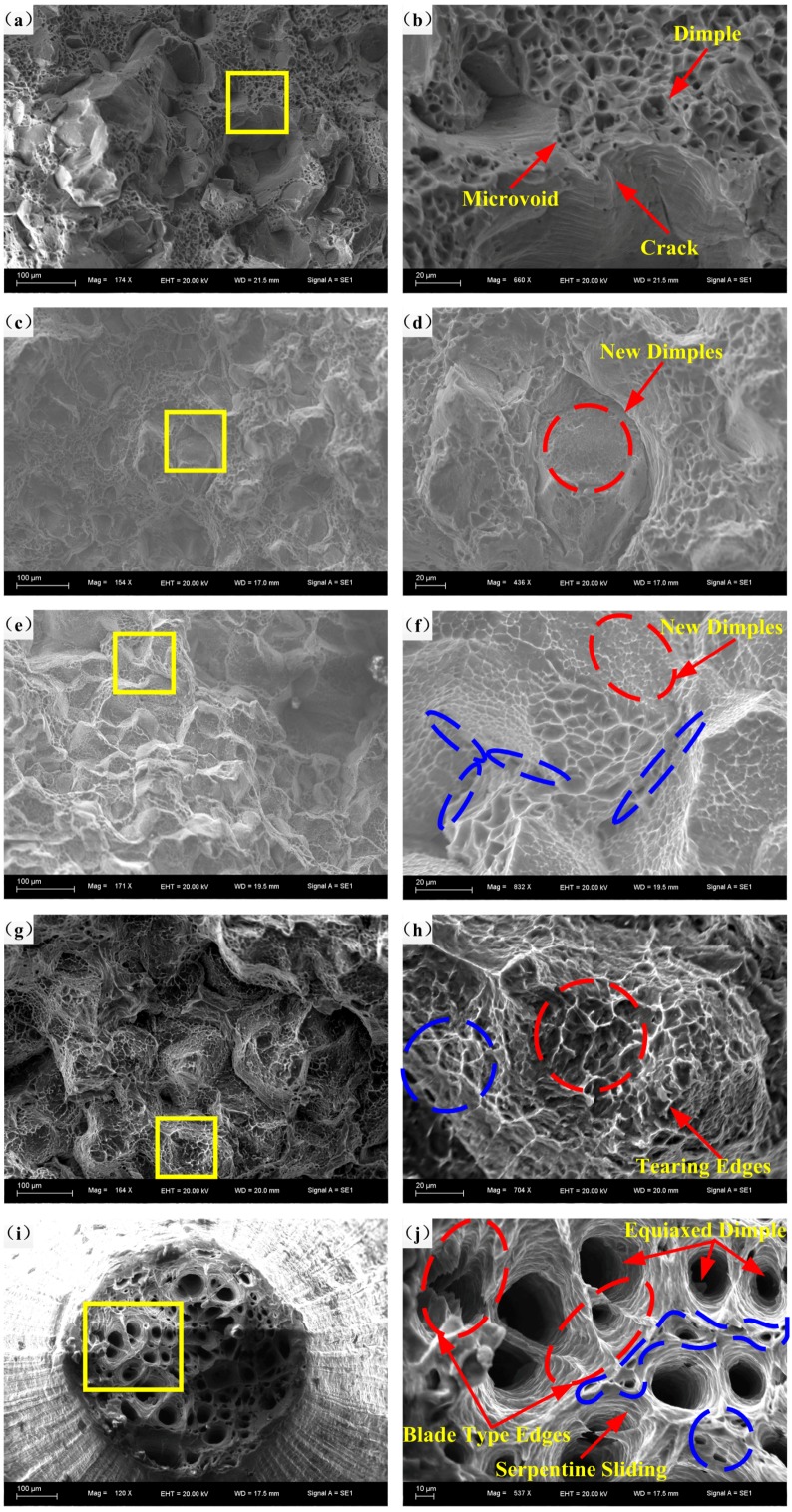
Fracture morphologies at strain rate of 10^−4^ s^−1^ and temperatures of: (**a**,**b**) RT; (**c**,**d**) 100 °C; (**e**,**f**) 200 °C; (**g**,**h**) 300 °C and (**i**,**j**) 400 °C.

Figure 10 depicts the fracture morphologies and corresponding high-resolution figures at the strain rate of 10^−4^ s^−1^and temperatures from RT to 400 °C. It is apparent that the effect of elevated temperature on the microstructure of specimens is remarkable, and we found that the evolution mechanism of the microstructure at each selected temperature range is of great significance. At RT, a mixed fracture mechanism of transgranular and intergranular facets is shown in Figure 10a. Small dimples, microvoids, and cracks can be observed (Figure 10b). At 100 °C, new dimples are generated on the intergranular facets (Figure 10c,d). At 200 °C, a small amount of microvoids has been found between intergranular facets, and the fracture surface is almost fully covered with a very high density of original and newly generated dimples (Figure 10e,f), but the coalescence of dimples cannot be observed. However, noticeable coalescence of dimples and microvoids, and remarkable tearing edges, can be observed at 300 °C (Figure 10h) signifying a localized melting mechanism and a more stable deformation process. Dimples become deeper and larger, and more microvoids appear on the fracture surface (Figure 10h), mainly due to higher diffusion rate and more active mobility of atoms caused by the elevation of temperature. The coalescence of dimples is likely to benefit the formation and propagation of large cracks during the tensile deformation process [1]. This phenomena also accords with the grain boundary mediated mechanisms resulting in higher ductility of rolled specimens with finer microstructure [30,31,32,33]. *Tm* is the melting temperature of alloy, and at temperatures above 0.5 *Tm*, (*Tm* = 475 °C), deformation led by grain boundary sliding and diffusion creep can result in a fine-grain-sized alloy, which is weaker than its counterpart at relatively low temperature with a large grain size [10]. Therefore, there exists a remarkable increasing of elongation to fracture and a reduction of peak stress at 300 °C. When temperature is elevated at 400 °C, the fracture surface is filled with a majority of equiaxed dimples and microvoids (Figure 10i), which can be concluded to be a ductile fracture mechanism. As depicted in Figure 10j, typical blade type edges can be found on the walls of large dimples and cracks, representing the coalescence of dimples in the hot tensile deformation process. On the inner surfaces of dimples, plenty of relatively smooth serpentine sliding can be observed, for the reason that new sliding occurs on free dimple surfaces along the direction of tension. Thus, it can be deduced that the specimen still suffers plastic deformation after the dimples form. Depth of dimples become deeper with the increase of temperature from RT to 400 °C (Figure 10), indicating dimples suffer elongation along the loading direction in deformation process. Shang, X. et al. [18] pointed out that voids suffer an evident elongation with the matrix along the loading direction in the deformation during the DRX process. Moreover, microstructures of fracture specimens from RT to 400 °C have been refined. The refining microstructure caused by DRX is able to improve the plastic deformation capacity of the material and increase the elongation to fracture (Figure 4 and Figure 10), which has been proposed and proved by other researchers [19]. DRX is more likely to happen under higher deformation temperature and lower strain rate [34,35]. To sum up, from RT to 400 °C, the quantity, size, and depth of dimples become larger and larger. Above 200 °C, the coalescence of dimples and microvoids, equiaxed dimples, and other typical structure have shown up, demonstrating that 200 °C is a transitional temperature.

#### 3.5.2. Effects of Strain Rate on Fracture Morphology

**Figure 11:**
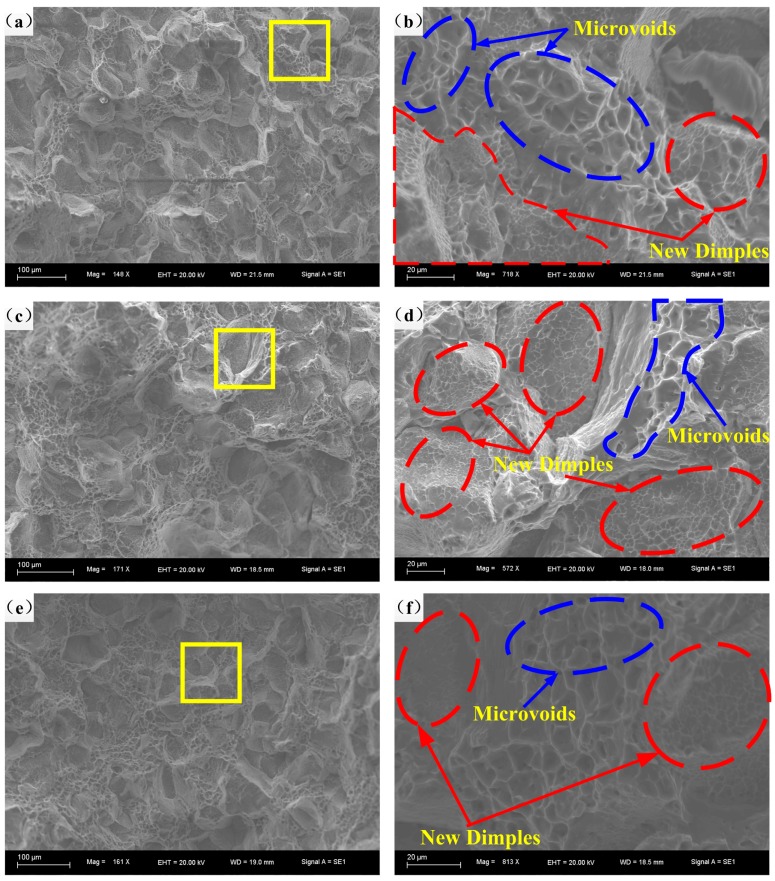
Fracture morphologies at 200 °C and strain rates of: (**a**,**b**) 10^−3^ s^−1^; (**c**,**d**) 10^−2^ s^−1^; (**e**,**f**) 10^−1^ s^−1^.

The fracture morphologies at 200 °C and 10^−3^–10^−1^ s^−1^and corresponding high-resolution figures are demonstrated in Figure 11. It is obvious that with the reduction of strain rate, there are more and more dimples and microvoids, because a lower strain rate provides more time for the accumulation of energy and the formation of dimples and microvoids. However, the size and depth of dimples are almost similar, and the coalescence of dimples and microvoids can hardly be observed. Taheri-Mandarjani, M. et al. [17] suggested that the internal necking of alloy is less likely to happen at relatively low temperature, and further straining is able to result in the generation of dimples instead of the enlargement of previous dimples.

**Figure 12:**
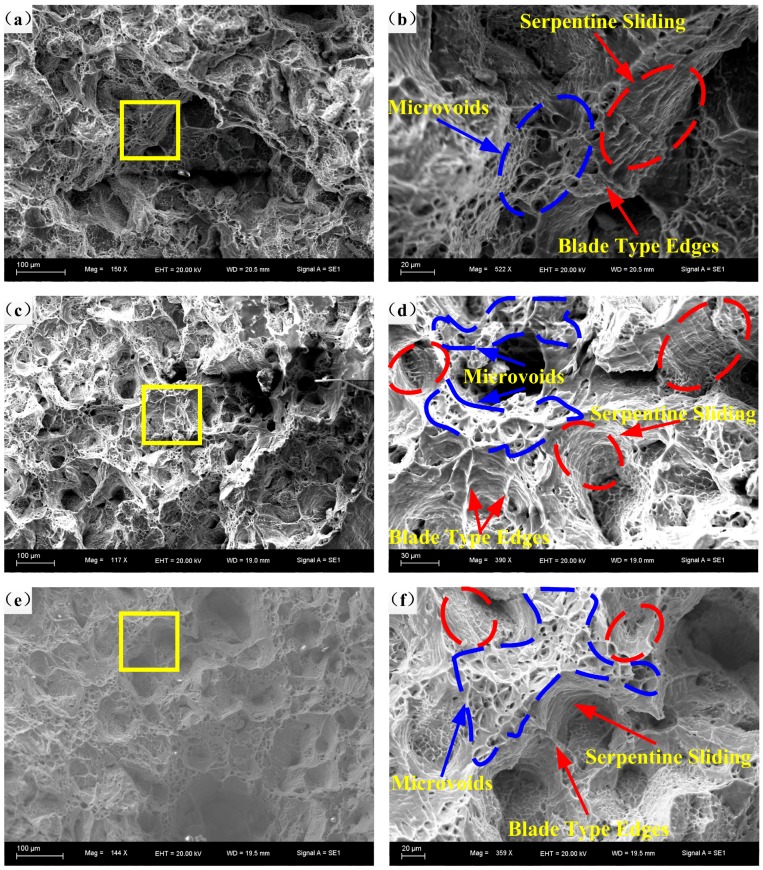
Fracture morphologies at 300 °C and at strain rates of: (**a**,**b**) 10^−3^ s^−1^; (**c**,**d**) 10^−2^ s^−1^; (**e**,**f**) 10^−1^ s^−1^.

Figure 12 depicts the fracture morphologies and their corresponding high resolution figures at 300 °C and 10^−3^–10^−1^ s^−1^. From Figure 10g,h and Figure 12, at 300 °C, it is apparent that the fracture surfaces are fully covered with dimples, microvoids, and noticeable coalescence of dimples. Typical blade type edges can be found on the walls of large dimples (Figure 12). Microstructures at various strain rates are different, with the decline of strain rate, the quantity of microvoids, and single dimples become less, and the phenomena of coalescence become more prominent. As shown in Figure 12b,d,f, from 10^−3^ to 10^−1^ s^−1^, blade type edges are blunter and less frequent. Moreover, on the inner surfaces of dimples, plenty of serpentine sliding can be observed, from which it can be inferred that the specimen still suffers plastic deformation after the dimples formed. Furthermore, ripples that are perpendicular to serpentine sliding can be found, which serves as evidence of the coalescence of small dimples during the plastic deformation process. This phenomena is more obvious with the increasing of strain rate. Until 10^−4^ s^−1^, blade type edges almost cannot be found, and the fracture surface is completely and evenly covered with the coalescence of dimples (Figure 10h). Thus, microstructures at 300 °C are more sensitive to the change of strain rate, which accords with macroscopic analyses mentioned above.

**Figure 13:**
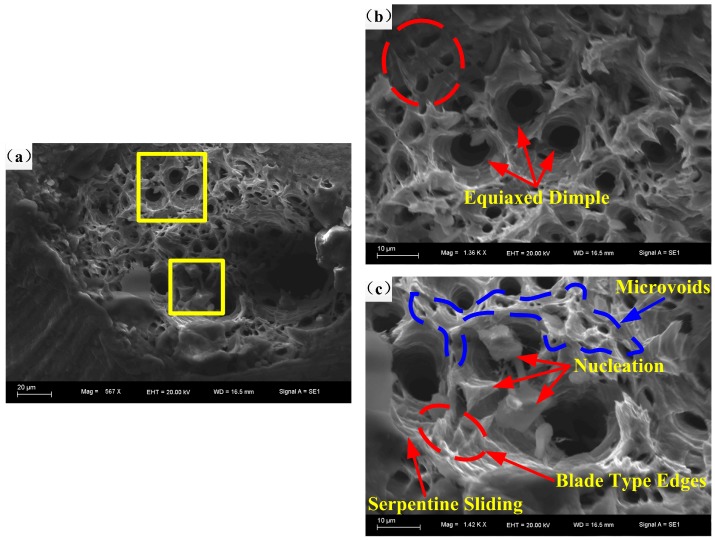
Fracture morphology at 400 °C and at strain rate of (**a**–**c**) 10^−1^ s^−1^.

Figure 13 shows the fracture morphology at 400 °C and 10^−1^ s^−1^. Figure 13b,c are high resolution figures denoted in Figure 13a. Typical equiaxed dimples, microvoids, blade type edges, serpentine sliding, and nucleation can be found on the fracture surface (Figure 13a,b). There are distinct equiaxed dimples, nucleation, coalescence of dimples, and more refined microstructures observable in Figure 13, which proves the occurrence of DRX [18]. Compared with Figure 10i,j, at 400 °C, the quantity, size, and depth of equiaxed dimples become larger with the reduction of strain rate. Besides, on the fracture surface of 10^−1^ s^−1^, there are more microvoids, sharper blade type edges, tearing edges, and a rougher surface of serpentine sliding, compared with its counterpart shown in Figure 10i,j. The phenomenon reveals the coupling effect of higher temperature and lower strain rate on the microstructure of the studied alloy, which is able to provide a heat environment for the activation of the thermal softening mechanism and more time for the accumulation of deformation energy. Then, the refining of microstructure can be achieved, which eventually leads to the increase of elongation to fracture and work of fracture, and the decrease of peak stress.

## 4. Conclusions

In the present work, effects of temperature and strain rate on the fracture behavior of an Al-Zn-Mg-Cu alloy are studied by uniaxial tensile tests at temperatures and strain rates from RT to 400 °C and from 10^−4^ s^−1^ to 10^−1^ s^−1^, respectively.

The elevation of temperature leads to the increasing of elongation to fracture and the reduction of peak stress, while higher strain rate results in the decreasing of elongation to fracture and the increasing of peak stress. *m*-Value considerably rises at 200 °C, and *W_f_* fluctuates drastically with the increase of strain rate at RT and 100 °C, which all signify a non-uniform and unstable deformation state below 200 °C. A competition of WH, DRV, and DRX exists at 200 °C, making it serve as a transitional temperature. Below 200 °C, WH is the main deformation mechanism of flow stress, and DRV and DRX dominates the flow stress above 200 °C.

The microstructure has been investigated by SEM. It has been found that from RT to 200 °C, the main feature of microstructure is the generation of dimples and microvoids. Above 200 °C, the coalescence of dimples and microvoids dominates the variation of microstructure, while the phenomenon of typical equiaxed dimples and nucleation appear at 400 °C. More importantly, microstructures are sensitive to various strain rates above 200 °C, which perfectly accords with macroscopic results.

The present work can provide a comprehensive understanding of flow stress of an Al-Zn-Mg-Cu alloy, and can offer valuable information to the optimization of the hot forming process and structural design of the studied alloy.

## References

[1] Zhou M., Lin Y.C., Deng J., Jiang Y.-Q. (2014). Hot tensile deformation behaviors and constitutive model of an Al–Zn–Mg–Cu alloy. Mater. Des..

[2] Shin D.H., Lee C.S., Kim W.J. (1997). Superplasticity of fine-grained 7475 Al alloy and a proposed new deformation mechanism. Acta Mater..

[3] Lee W.S., Sue W.C., Lin C.F., Wu C.-J. (2000). The strain rate and temperature dependence of the dynamic impact properties of 7075 aluminum alloy. J. Mater. Process. Technol..

[4] Voyiadjis G.Z., Abed F.H. (2005). Microstructural based models for bcc and fcc metals with temperature and strain rate dependency. Mech. Mater..

[5] Wang L., Yu H., Lee Y., Kim H.-W. (2015). Hot tensile deformation behavior of twin roll casted 7075 aluminum alloy. Met. Mater. Int..

[6] Picu R.C., Vincze G., Ozturk F., Gracio J.J., Barlat F., Maniatty A.M. (2005). Strain rate sensitivity of the commercial aluminum alloy AA5182-O. Mater. Sci. Eng. A.

[7] Srivatsan T.S., Guruprasad G., Vasudevan V.K. (2008). The quasi static deformation and fracture behavior of aluminum alloy 7150. Mater. Des..

[8] Zhang H., Jin N.P., Chen J.H. (2011). Hot deformation behavior of Al-Zn-Mg-Cu-Zr aluminum alloys during compression at elevated temperature. Trans. Nonferrous Met. Soc. China.

[9] Deng J., Lin Y.C., Li S.S., Chen J., Ding Y. (2013). Hot tensile deformation and fracture behaviors of AZ31 magnesium alloy. Mater. Des..

[10] Lin Y.C., Deng J., Jiang Y.Q., Wen D.-X., Liu G. (2014). Effects of initial δ phase on hot tensile deformation behaviors and fracture characteristics of a typical Ni-based superalloy. Mater. Sci. Eng. A.

[11] Shojaei K., Sajadifar S.V., Yapici G.G. (2016). On the mechanical behavior of cold deformed aluminum 7075 alloy at elevated temperatures. Mater. Sci. Eng. A.

[12] Mishra R.S., Mahoney M.W., McFadden S.X., Mara N.A., Mukherjee A.K. (1999). High strain rate superplasticity in a friction stir processed 7075 Al alloy. Scr. Mater..

[13] Lin Y.C., Chen M.S., Zhong J. (2008). Microstructural evolution in 42CrMo steel during compression at elevated temperatures. Mater. Lett..

[14] Cerri E., Evangelista E., Forcellese A., McQueen H.J. (1995). Comparative hot workability of 7012 and 7075 alloys after different pretreatments. Mater. Sci. Eng. A.

[15] Lin Y.C., Chen X.M. (2011). A critical review of experimental results and constitutive descriptions for metals and alloys in hot working. Mater. Des..

[16] El-Magd E., Abouridouane M. (2006). Characterization, modelling and simulation of deformation and fracture behaviour of the light-weight wrought alloys under high strain rate loading. Int. J. Impact Eng..

[17] Taheri-Mandarjani M., Zarei-Hanzaki A., Abedi H.R. (2015). Hot ductility behavior of an extruded 7075 aluminum alloy. Mater. Sci. Eng. A.

[18] Shang X., Cui Z., Fu M.W. (2017). Dynamic recrystallization based ductile fracture modeling in hot working of metallic materials. Int. J. Plast..

[19] Sunter B.J., Burman N.M. (1972). Development of Improved Hot Workability of Some Cu Alloys. J. Aust. Inst. Met..

[20] Lin Y.C., Chen M.S., Zhong J. (2009). Effects of deformation temperatures on stress/strain distribution and microstructural evolution of deformed 42CrMo steel. Mater. Des..

[21] Azarbarmas M., Aghaie-Khafri M., Cabrera J.M., Calvo J. (2016). Dynamic recrystallization mechanisms and twining evolution during hot deformation of Inconel 718. Mater. Sci. Eng. A.

[22] Lin Y.C., Dong W.Y., Zhou M., Wen D.-X., Chen D.-D. (2018). A unified constitutive model based on dislocation density for an Al-Zn-Mg-Cu alloy at time-variant hot deformation conditions. Mater. Sci. Eng. A.

[23] Momeni A., Kazemi S., Bahrani A. (2013). Hot deformation behavior of microstructural constituents in a duplex stainless steel during high-temperature straining. Int. J. Miner. Metall. Mater..

[24] Wu H., Zhang H., Shuang C., Fu D. (2015). Flow stress behavior and processing map of extruded 7075Al/SiC particle reinforced composite prepared by spray deposition during hot compression. Trans. Nonferrous Met. Soc. China.

[25] Peng J., Li K.S., Pei J.F., Zhou C.-Y. (2018). Temperature-Dependent SRS Behavior of 316L and Its Constitutive Model. Acta Metall. Sin. (Engl. Lett.).

[26] Fong K.S., Danno A., Tan M.J., Chua B.W. (2017). Tensile flow behavior of AZ31 magnesium alloy processed by severe plastic deformation and post-annealing at moderately high temperatures. J. Mater. Process. Technol..

[27] Yang Y., Zhao Y., Kai X., Tao R. (2017). Superplasticity behavior and deformation mechanism of the in-situ Al3Zr/6063Al composites processed by friction stir processing. J. Alloys Compd..

[28] Rodriguez A.K., Ayoub G.A., Mansoor B., Benzerga A.A. (2016). Effect of strain rate and temperature on fracture of magnesium alloy AZ31B. Acta Mater..

[29] Martin J.W., Doherty R.D., Cantor B. (1997). Stability of Microstructure in Metallic Systems.

[30] Montheillet F., Piot D., Matougui N., Fares M.L. (2014). A critical assessment of three usual equations for strain hardening and dynamic recovery. Metall. Mater. Trans. A.

[31] Wen D.X., Lin Y.C., Chen J., Chen X.-M., Zhang J.-L., Liang Y.-J., Li L.-T. (2015). Work-hardening behaviors of typical solution-treated and aged Ni-based superalloys during hot deformation. J. Alloys Compd..

[32] Puchi-Cabrera E.S., Guérin J.D., Barbier D., Dubar M., Lesage J. (2013). Plastic deformation of structural steels under hot-working conditions. Mater. Sci. Eng. A.

[33] Abbasi S.M., Morakkabati M., Sheikhali A.H., Momeni A. (2014). Hot deformation behavior of beta titanium Ti-13V-11Cr-3Al alloy. Metall. Mater. Trans. A.

[34] Chen X.M., Lin Y.C., Wen D.X., Zhang J., He M. (2014). Dynamic recrystallization behavior of a typical nickel-based superalloy during hot deformation. Mater. Des..

[35] Rokni M.R., Zarei-Hanzaki A., Roostaei A.A., Abedi H.R. (2011). An investigation into the hot deformation characteristics of 7075 aluminum alloy. Mater. Des..

